# The evolutionary history of LysM-RLKs (LYKs/LYRs) in wild tomatoes

**DOI:** 10.1186/s12862-019-1467-3

**Published:** 2019-07-11

**Authors:** Sarah Richards, Laura E. Rose

**Affiliations:** 1 Institute of Population Genetics, Heinrich Heine University, Duesseldorf, Universitaetsstr. 1, 40225 Duesseldorf, Germany; 2 CEPLAS, Cluster of Excellence in Plant Sciences, Heinrich Heine University, Duesseldorf, Universitaetsstr. 1, 40225 Duesseldorf, Germany

**Keywords:** Phylogenetics, Population genetics, Solanum, Plant immunity, Symbiosis

## Abstract

**Background:**

The LysM receptor-like kinases (LysM-RLKs) are important to both plant defense and symbiosis. Previous studies described three clades of LysM-RLKs: LysM-I/LYKs (10+ exons per gene and containing conserved kinase residues), LysM-II/LYRs (1–5 exons per gene, lacking conserved kinase residues), and LysM-III (two exons per gene, with a kinase unlike other LysM-RLK kinases and restricted to legumes). LysM-II gene products are presumably not functional as conventional receptor kinases, but several are known to operate in complexes with other LysM-RLKs. One aim of our study was to take advantage of recently mapped wild tomato transcriptomes to evaluate the evolutionary history of LysM-RLKs within and between species. The second aim was to place these results into a broader phylogenetic context by integrating them into a sequence analysis of LysM-RLKs from other functionally well-characterized model plant species. Furthermore, we sought to assess whether the Group III LysM-RLKs were restricted to the legumes or found more broadly across Angiosperms.

**Results:**

Purifying selection was found to be the prevailing form of natural selection within species at LysM-RLKs. No signatures of balancing selection were found in species-wide samples of two wild tomato species. Most genes showed a greater extent of purifying selection in their intracellular domains, with the exception of *Sl*LYK3 which showed strong purifying selection in both the extracellular and intracellular domains in wild tomato species. The phylogenetic analysis did not reveal a clustering of microbe/functional specificity to groups of closely related proteins. We also discovered new putative LysM-III genes in a range of Rosid species, including *Eucalyptus grandis*.

**Conclusions:**

The LysM-III genes likely originated before the divergence of *E. grandis* from other Rosids via a fusion of a Group II LysM triplet and a kinase from another RLK family. *Sl*LYK3 emerges as an especially interesting candidate for further study due to the high protein sequence conservation within species, its position in a clade of LysM-RLKs with distinct LysM domains, and its close evolutionary relationship with LYK3 from *Arabidopsis thaliana*.

**Electronic supplementary material:**

The online version of this article (10.1186/s12862-019-1467-3) contains supplementary material, which is available to authorized users.

## Background

Plants are regularly targeted for colonization by organisms ranging from pathogenic to beneficial [1]. Pathogenic organisms infect the plant and use it for nourishment, eventually reducing host fitness [1]. Pathogens can induce changes in the host plant’s genetic regulation to maximize the amount of nutrients that can be accessed and to avoid detection and subsequent host defense responses [1]. Beneficial symbiotic organisms also use the host plant for nourishment but offer benefits to the plant in exchange (e.g. better uptake of water and nutrients) [1]. Plants that can differentiate between pathogenic and beneficial symbiotic organisms improve their chances of survival and reproduction [1]. Detection of the presence of these organisms involves extracellular receptors, often receptor-like kinases (RLKs), which recognize proteins or other molecular “patterns” produced by the colonizing organisms and trigger phosphorylation and downstream signaling cascades within the cell [1, 2]. The signaling cascades then activate the appropriate defensive or symbiotic responses [2].

Genes containing the LysM motif, including the family of LysM-RLKs, have been implicated in the detection of both plant-symbiotic and -pathogenic organisms [3]. In the case of beneficial symbiotic organisms, some LysM-RLKs are the key coordinators of the plant’s cooperative response with the symbiont [3]. Other LysM-RLKs are necessary for detecting pathogens and activating defense responses. These responses can be initiated by the recognition of pathogen-derived molecules such as chitin as it is shed by the invading pathogen [3]. LysM-RLKs sometimes function together as heterodimers, with the extracellular region of one LysM-RLK detecting the presence of the colonizing pathogen or symbiont while the kinase domain of another LysM-RLK mediates the symbiotic or defense response [3, 4]. Some LysM-RLKs, such as *Oryza sativa* CERK1 (*Os*CERK1), also function as dual-purpose detectors of both pathogenic and symbiotic organisms [5]. It is not clear whether genes that play similar functional roles are more closely related to one another or if convergent evolution in microbe discrimination is widespread in this protein family.

LysM-RLKs, with three repetitions of the LysM domain, are ubiquitous among land plants [6] and may have evolved as part of a signaling module prior to colonization of land and the origin of symbiosis with mycorrhiza [7]. Shiu et al. describe two main clades of LysM-RLKs: LysM-I and LysM-II [8], with LysM-RLKs in Group II lacking conserved kinase residues; for example, the glycine-rich loop is missing from the kinase domains of all Group II LysM-RLKs from *Arabidopsis thaliana* and *Solanum lycopersicum* [6, 9]. LysM-I RLKs have ten or more exons, while LysM-II RLKs typically have one or two [6]. Another group consisting of four LysM-RLK genes (two from *Medicago truncatula* and two from *Lotus japonicus*) contain classically conserved kinase residues (like LysM-I RLKs) and two exons (like LysM-II RLKs). However, due to their structural similarities to both groups, these genes cannot be classified unambiguously into either Group I or Group II, and their clade has been named Group III [10, 11]. Arrighi et al. have suggested that the Group III LysM-RLKs *Mt*LYR5 and *Mt*LYR6 are of chimeric origin, arising from the fusion of one gene encoding a LysM triplet and a second gene encoding a kinase domain, unlike those found in other LysM genes [10]. Lohmann et al. came to the same conclusion based on their analyses of the only other characterized Group III genes (found in *L. japonicus*) and further suggested that Group III arose within the dicot lineage [11]. Until now, Group III genes have not been reported from outside the Leguminosae. Furthermore, although phylogenetic analyses of LysM-RLKs from a variety of plant species have been conducted, a comprehensive phylogenetic analysis of this family across several species (including tomato) is lacking. Simultaneously, the availability of newly-described functions of individual LysM-RLKs from well-studied species provided an opportunity to evaluate the distribution of functional specificity in a phylogenetic context.

Here we synthesize the currently available information about function (i.e. microbe specificity) and phylogenetic relationships of LysM-RLKs, including those from tomato, and show a close relationship between Group II and Group III LysM-RLKs. Newly discovered putative Group III LysM-RLKs are present in a wide variety of Rosid species. However, they were not detected outside the Rosid clade. Orthologs of *Sl*LYK3 show evidence of strong purifying selection in wild tomatoes, and although the kinase domain of *Sl*LYK8 is truncated in cultivated tomato, we find that this is not the case for orthologs in wild tomato species.

## Results

### Distribution of microbe specificity across the phylogenetic tree

**Fig. 1:**
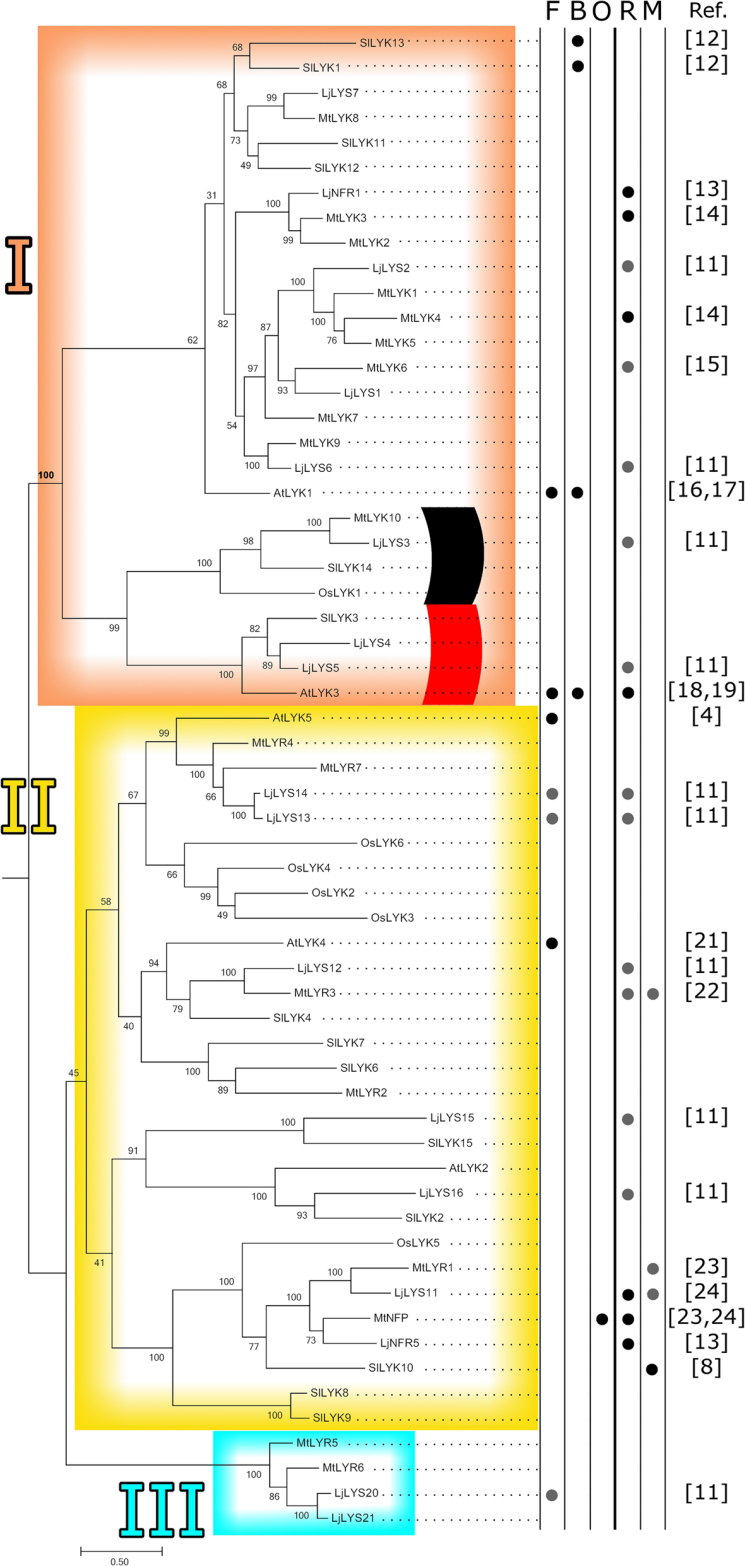
LysM-RLK phylogeny and functions. Maximum likelihood phylogeny and functions of canonical LysM-RLKs from *Solanum lycopersicum* (*Sl*), *Arabidopsis thaliana* (*At*), *Lotus japonicus* (*Lj*), *Oryza sativa* (*Os*), and *Medicago truncatula* (*Mt*). The phylogeny and 500 bootstrap replicates were inferred using RAxML under the WAG model with empirical frequencies and seed values of 100. The species of gene origin is given by the first two letters of the name given on the phylogeny. The phylogeny was rooted using the method of Minimal Ancestor Deviation [27]. The scale bar indicates amino acid substitutions per site. Gene functions are indicated: defense against fungi (F), bacteria (B), and oomycetes (O) and symbiosis with rhizobia (R) and mycorrhiza (M). LysM-RLKs form three clades. Red and black arcs indicate groups of proteins with distinct LysM domain sequences. Functions verified by mutation phenotypes are indicated by black circles. Functions inferred by differential expression are indicated by gray circles. Citations for sources of functional information are shown in brackets

The goal of this study was to understand the evolutionary history of the LysM-RLKs on two levels: at the microevolutionary scale (within the clade of wild tomatoes) and at the macroevolutionary scale. At the larger evolutionary time scale, understanding the broader evolutionary patterns, especially in terms of microbe recognition, will allow us to place the insights gained from the microevolutionary analysis in context. We aimed to test if LysM-RLKs with similar microbial recognition specificity clustered phylogenetically. We assembled a large set of all previously defined (canonical) LysM-RLKs reported from species for which the greatest amount of functional data was available: *A. thaliana*, *L. japonicus*, *M. truncatula, O. sativa*, and *S. lycopersicum* [4, 9, 11, 17, 33–45]. We then aligned the LysM-RLK protein sequences using MUSCLE, inferred the phylogenetic relationships and combined it with the known functions of the proteins (Fig. 1). The maximum-likelihood phylogeny was rooted using MAD [27], a modified midpoint rooting method that takes lineage-specific heterotachy into account. This makes this method robust to variation in evolutionary rates among lineages and allows rooting without a priori determination of an outgroup. We differentiated between functions inferred by correlated gene expression/regulation patterns and those established by analyses of mutant phenotypes. This was an important distinction to make, because it is possible for a gene to be co-regulated upon microbe exposure without the gene necessarily playing a role in symbiosis or defense (e.g. *Lj*LYS11) [43]. While we do recover multiple small clades of closely related sequences reported to fulfill similar functions, in most cases, major clades do not appear to be strictly associated with a specific form of microbe recognition. This suggests that microbe recognition can evolve convergently throughout the family and that orthologous genes can encode for different recognition specificities. This agrees with the results of de Mita et al. who showed neofunctionalization among LysM-RLK gene duplicates [46]. However, it should be noted that most of the genes have not necessarily been tested for each of the functions listed, and functions in the best-studied functional process - Rhizobia symbiosis – dominate the tree. This bias in functional characterization may limit our power to detect a strong correlation between the type of microbe recognition and phylogenetic position.

### Group III LysM-RLKs cluster with group II LysM-RLKs

The relationships between the major gene groups are generally consistent with those of previously published phylogenies based on entire coding regions or kinase domains of a subset of these LysM-RLKs (Fig. 1; references [9, 12]). We observe a separation of the LysM-RLKs into two major clades: Group I sequences in one clade and Group II and Group III sequences in a second clade. The monophyly of these clades is well-supported (100% bootstrap support, bold on Fig. 1), assuming correct rooting of the phylogeny. Furthermore, the genes of Group I are further differentiated into two subclades. These genes differ by the presence of a microexon in one group and the absence of this exon in the other group, as previously reported by Lohmann and colleagues [11]. According to our analysis, Group II and Group III share a more recent common ancestor than either does with Group I.

### Group III LysM-RLKs exist in diverse Rosid species

Group III LysM-RLKs were previously reported only from *L. japonicus* and *M. truncatula*. To determine whether Group III LysM-RLKs are present outside of the Leguminosae and specifically in tomato, we searched NCBI’s non-redundant protein database for homologs of *Lj*LYS20 using BlastP with a limit of 250 hits. This produced as many putative Group III LysM-RLKs as obtained by increasing the search limit to 500 hits or by using each of the four known Group III LysM-RLKs (*Lj*LYS20, *Lj*LYS21, *Mt*LYR5 and *Mt*LYR6) and merging the results. The 250 hits aligned to the query by BLAST were at least 42% identical to the query sequence (679 amino acids). The majority of candidate sequences identified in the search originated from legume species, but some came from additional species across the Rosid clade. No sequences were found in the Asterids, the clade containing the tomatoes. To determine if these new sequences were bona fide Group III LysM-RLKs, we determined whether they contained the highly-conserved CXC motif between the 1st and 2nd LysM domains. From the GUIDANCE alignment of these sequences, we determined that 117 of the 250 hits had the defining CXC motif.

To determine whether additional putative Group III LysM-RLKs may have been missed in the BlastP search, an online psiBLAST [47] with LjLYS20 as query was performed. Five iterations of psiBLAST [47] were run, under standard settings and using an E-value cut-off of 0.05. Only the portions of the sequences which aligned to the query were downloaded. Since the CXC motif occurs at positions 109–111 of *Lj*LYS20, the first 130 amino acids of the hits were aligned with the canonical Group III LysM-RLKs using MUSCLE to check for the presence of the CXC motif. The second psiBLAST iteration produced some sequences with CXC motifs, but alignment with the canonical LysM-RLKs revealed that they did not have LysM domains and were not part of the LysM-RLK family. The third iteration produced a sequence with CXC motif which closely resembled another sequence from the same species in the original BLASTp search. The fourth and fifth iterations produced no sequences with CXC motifs. We concluded that the psiBLAST search did not result in additional putative Group III LysM-RLKs and that the BlastP search was sufficiently exhaustive. We included the 117 unique putative Group III sequences from the BlastP search in our phylogenetic analysis of the canonical LysM-RLKs.

**Fig. 2:**
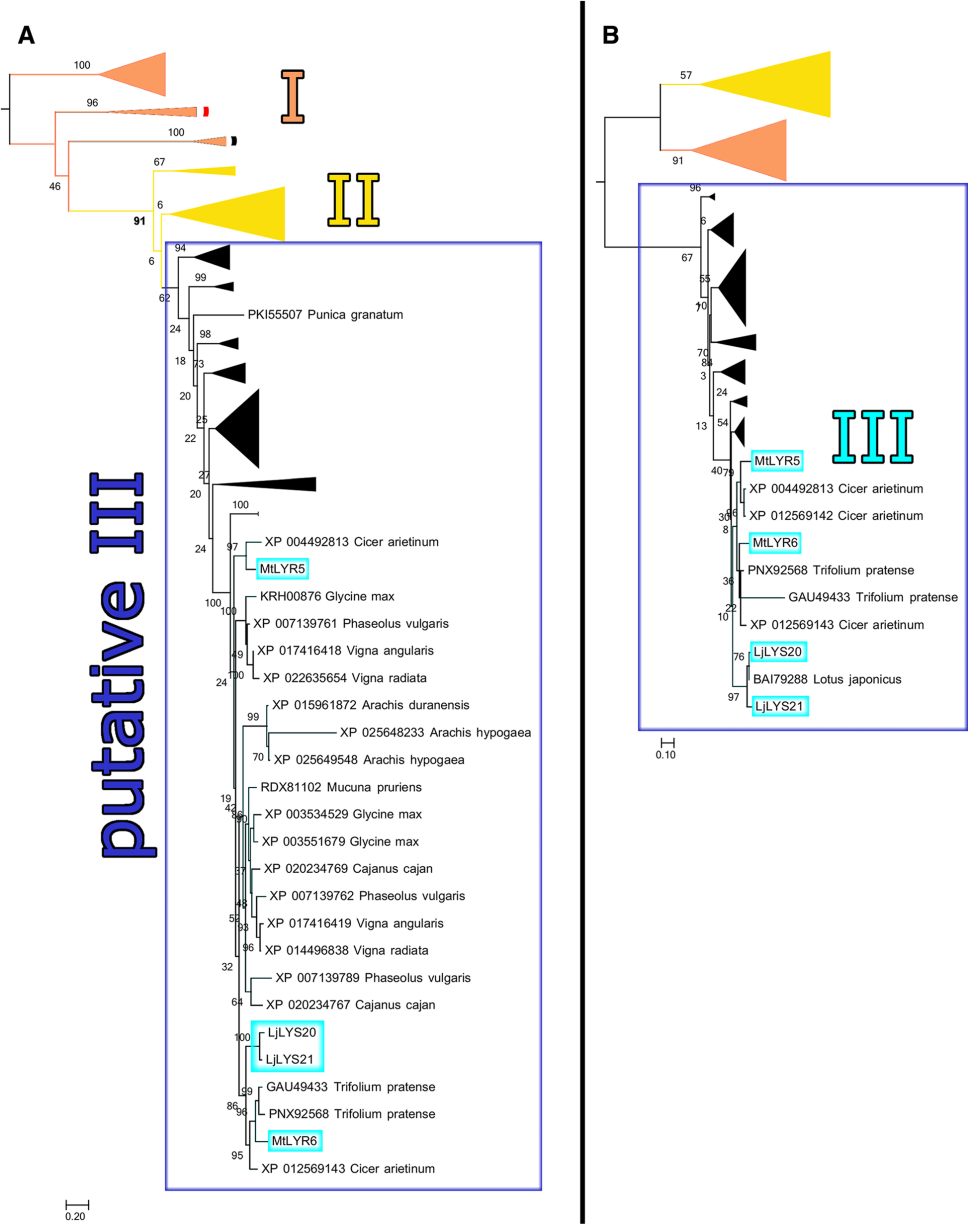
Phylogeny of canonical LysM-RLKs and CXC-motif-containing BlastP hits of *Lj*LYS20. **a** Phylogeny of the LysM domain sequences only shows Group III and putative Group III sequences forming a clade with Group II. The phylogeny and 500 bootstrap replicates were inferred using RAxML under the WAG model with empirical frequencies and seed values of 100 and rooted using the method of Minimal Ancestor Deviation [27]. **b** Phylogeny of the kinase domains only shows Group I and Group II forming a clade together, while Group III and putative Group III sequences form another clade. The phylogeny and 500 bootstrap replicates were inferred using RAxML under the JTT model with empirical frequencies and seed values of 100 and rooted using the method of Minimal Ancestor Deviation [27]. The scale bar indicates amino acid substitutions per site

In the combined analysis, the canonical Group III members (*Lj*LYS20, *Lj*LYS21, *Mt*LYR5 and *Mt*LYR6) and new putative Group III LysM-RLK genes form a clade together (Additional files 3 and 4). In agreement with our initial analysis of the canonical LysM-RLKs (Fig. 1), we observe that Group II and Group III share a more recent common ancestor with one another than either does with Group I. However, all Group II genes lack conserved kinase residues, while Group III genes encode a kinase *unlike* other LysM-RLK kinases [10, 11]. Since the extracellular LysM region and intracellular kinase region of Group III genes may have different ancestries due their putative chimeric origin [10, 11], we inferred the phylogenetic history of these two regions separately (Fig. 2, Additional files 5 and 6).

If these newly identified sequences were true homologs of the four original Group III sequences, we expected Group II sequences to form a clade together with the original and new Group III sequences in analyses based on the LysM domain region. As before, we used the MAD method to root these phylogenies. We recovered a clade containing the Group II genes along with the new and canonical Group III genes with 91% bootstrap support in our analysis of the LysM domain region of these genes (bold in Fig. 2a, Additional file 5). In contrast, a phylogeny based on the kinase domain would be expected to include both the new and previously described Group III genes in a separate monophyletic clade from Group I and II genes and this is what we observe (Fig. 2b, Additional file 6).

The new Group III LysM-RLKs in our analysis indicate a wider taxonomic distribution of Group III genes in species outside the Leguminosae. These new gene sequences are found in species throughout the Rosids, but not from the Asterids (which includes tomatoes) or other Eudicots. This is consistent with Group III genes arising relatively early in the Rosid lineage. Nevertheless, their presence exclusively in the Rosid lineage implies that they arose after the monocot/dicot split. In contrast, since Group I and Group II genes are found in both monocots and dicots, these genes likely originated and began to diverge prior to the monocot/dicot split. The sequence similarity in the LysM domain region between Group III and Group II genes implies that a Group II ancestral gene was the likely donor of the LysM triplet at the time of origin of Group III genes in Rosids.

### The LysM domains of *Sl*LYK3, *At*LYK3, *Lj*LYS4, and *Lj*LYS5 are distinct from other LysM-RLKs

The ability of LysM-RLK proteins to detect and distinguish between ligands depends on their three extracellular LysM domains. Therefore, the evolutionary history of this region is especially relevant for understanding how functional differences arise and are maintained. Based on previous functional studies, the LysM2 domain of certain LysM-RLKs has emerged as the most critical for ligand recognition and discrimination [48]. Therefore, we were interested in uncovering the evolutionary history of each LysM domain in isolation and whether individual domains showed differential patterns of association with functional recognition. To this end, we sought to reconstruct the evolutionary history of the LysM1, LysM2, and LysM3 domains from the set of canonical LysM-RLK genes of *S. lycopersicum*, *A. thaliana*, *L. japonicus*, *M. truncatula*, and *O. sativa*.

**Fig. 3:**
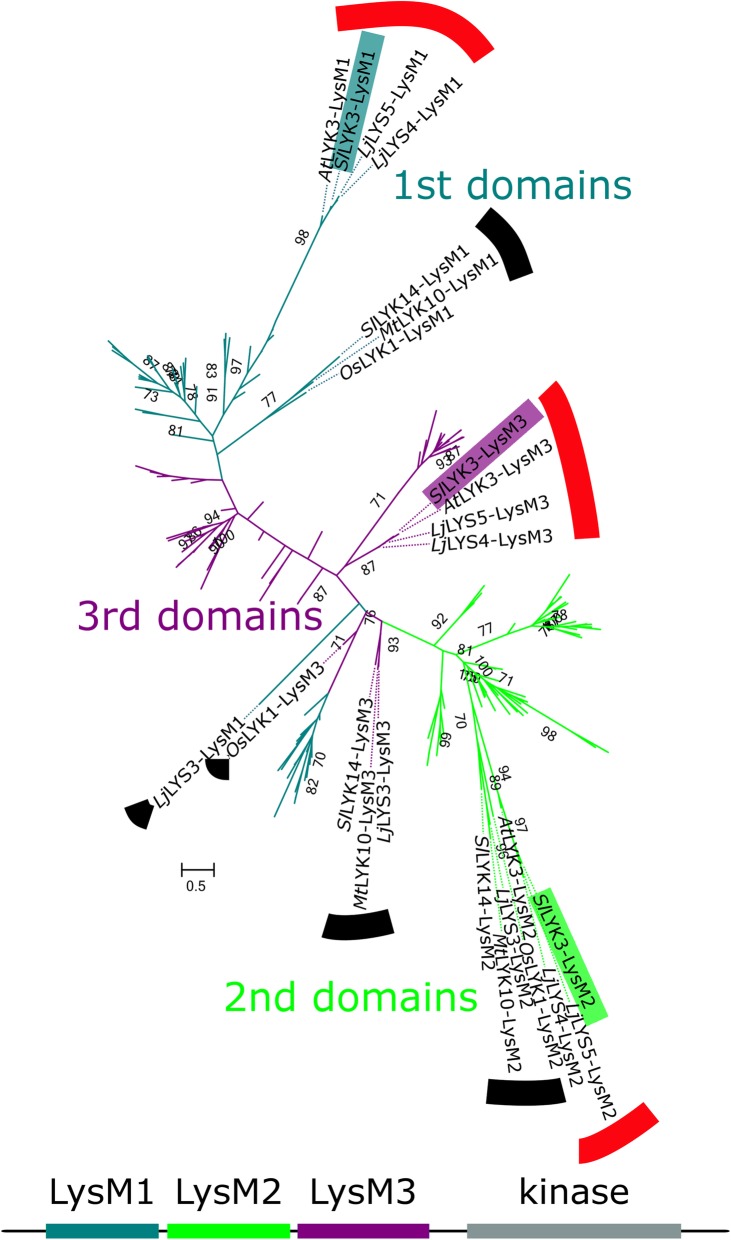
Unrooted phylogeny of individual LysM-RLK domains. Phylogeny of amino acid sequences of each of the three LysM-RLK protein domains from each of the canonical LysM-RLKs of *Solanum lycopersicum* (*Sl*), *Arabidopsis thaliana* (*At*), *Lotus japonicus* (*Lj*), *Oryza sativa* (*Os*), and *Medicago truncatula* (*Mt*). Each gene is represented three times in the tree, once for each individual LysM domain (see emphasis on *Sl*LYK3 in phylogeny), with color-coding by domain position. The phylogeny and 500 bootstrap replicates were inferred using RAxML under the WAG model with empirical frequencies and seed values of 100. The Log-likelihood was −13,081. The scale bar indicates amino acid substitutions per site. Sequences from the first, second, and third LysM domains generally cluster in clades with others of the corresponding LysM domain, but the first and third domains do not form separate clades. Especially long branches subtend the groups of the first and second domains of genes of interest, referred to as the red clade and the black clade. Domain sequences from the red clade are highlighted in red: *At*LYK3, *Sl*LYK3, *Lj*LYS4, and *Lj*LYS5. Those from the black clade are highlighted in black: *Os*LYK1, *Mt*LYK10, *Sl*LYK14, and *Lj*LYS3. The third domain of *Os*LYK1 and first domain of *Lj*LYS3 are separated from the corresponding domains of the second group

The short sequence lengths (about 60 amino acids) and substantial variation between the individual LysM domain sequences led to poor phylogenetic resolution. Since the three-LysM-domain structure is ancient [3], we expected to recover a tree with three distinct clades consisting of sequences from the first, second and third domains, respectively. However, our phylogenetic analysis did not show three monophyletic clades according to domain order (Fig. 3, Additional file 7). Instead resolution, especially between the first and third domain sequences, was exceptionally low. Furthermore, branches subtending some lineages were substantially longer than others. The lineages containing sequences from four genes in particular, *Sl*LYK3, *At*LYK3, *Lj*LYS4, and *Lj*LYS5 (indicated in red swatches in Figs. 1 and 3 and referred to subsequently as the “red clade”), stood out for each LysM domain and consistently formed clades supported by bootstrap values near 90%.

We suspected that the long branches subtending these clades could indicate a problem with the alignment of these sequences. A GUIDANCE [25] analysis indicated that for the third domain, similar alignments resulted independent of alignment method. For the first and second domains, different alignments were recovered depending upon which alignment method was used.

In particular, the GUIDANCE sequence score was less than 0.8 for the alignments of the first domains of all of the proteins, as well as the second domains of the previously described group *Sl*LYK3, *At*LYK3, *Lj*Lys4, and *Lj*LYS5 (the red clade) and another group *Os*LYK1, *Sl*LYK14, *Lj*LYS3, and *Mt*LYK10 (indicated by a black swatch in Figs. 1 and 3 and referred to as the “black clade”). We conclude that the phylogenetic signal in these data is very weak due to extensive sequence divergence.

**Fig. 4:**
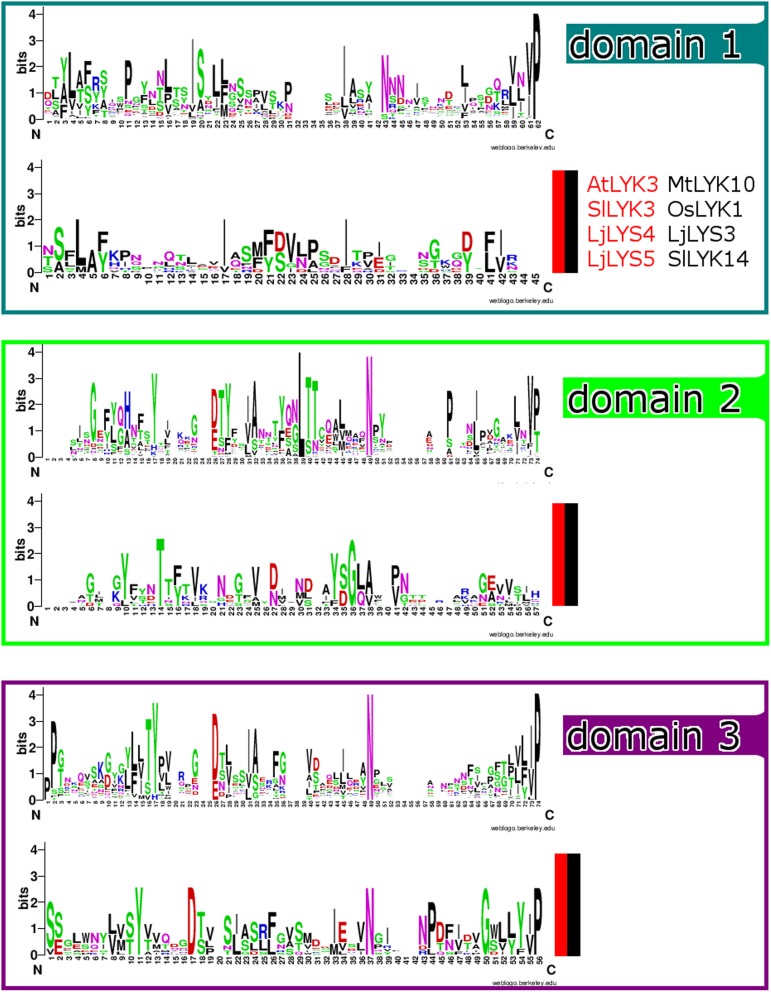
Amino acid logos of LysM-RLK domains. Logos of LysM-RLK LysM domains, with those of the red and black clades (*At*LYK3, *Sl*LYK3, *Lj*LYS4, *Lj*LYS5, *Mt*LYK10, *Os*LYK1, *Lj*LYS3, and *Sl*LYK14) computed separately. The third domains of both sets of sequences share conserved amino acids, while first and second domains of the two sequence sets share few conserved amino acids

This analysis prompted us to inspect the degree to which residues of the individual LysM domains were conserved. Also we investigated whether the LysM domain sequences from the genes belonging to the red and black clades had substantially diverged from the other genes, especially at residues otherwise conserved across genes. The WebLogo [49] analysis of the sequences for the three individual LysM domains, excluding the sequences belonging to the red and black clades, showed that the conservation of individual residues varied across all three LysM domains (Fig. 4). A moderate number of conserved residues (four) were shared between both groups of LysM-RLKs in the third LysM domain (Fig. 4). However, the amino acid logos for the LysM domain sequences from the red and black clades show nearly no overlap with the conserved residues in LysM domains 1 and 2. In summary, the representative genes of the red and black clades are evolutionarily divergent compared to those from other LysM-RLK genes, showing nearly no overlap in conserved residues in LysM domains 1 and 2. This is further indicated by their position on the phylogeny based on the entire protein sequences (Fig. 1): There it is noted that genes from the red and black clades belong to Group I and form a well-supported monophyletic sister clade to the other members of the Group I LysM-RLKs.

Based upon the outcome of the Logo analysis and GUIDANCE, we reanalyzed the individual LysM domains for the gene and domain combinations that could be confidently aligned (Additional files 8 and 9). For this analysis, red and black clade domain 2 sequences and all sequences from domain 1 were excluded due to low confidence in the alignments (as described above). The resulting phylogeny shows good resolution between domain 2 and 3 sequences (bootstrap support of 89%, bold on Additional file 8), consistent with the maintenance of this domain structure tracing back at least to the split of monocots and dicots.

### Purifying selection is the prevalent form of natural selection in LysM-RLKs from wild tomatoes

To evaluate the evolutionary history of the LysM-RLKs on a more recent microevolutionary timescale, we investigated the patterns of polymorphism and divergence in LysM-RLK genes in a young pair of wild tomato species, *S. chilense* and *S. peruvianum*. We first calculated standard population genetic summary statistics for these genes (Table 1) in the species of interest. LysM-RLK sequences from *S. ochranthum* and *S. lycopersicoides* were used as outgroups. These are ideal outgroups. Their evolutionary position is outside our clade of interest, which itself also includes the cultivated tomato, *S. lycopersicum* [20]; these two outgroup species are evolutionarily closer to the wild tomatoes than is *S. tuberosum* (potato); and both outgroup species are diploids, rather than polyploids.

**Table 1:** Summary of allelic diversity and tests of neutrality of LysM-RLKs in wild tomatoes

	No. Seqs	No. Sites	Haplotypes	π all sites (non,syn,silent)	*π*_*a*_/*π*_*s*_	S	MK *p*-value (uncorrected)
LYK1		1881					
chil v ochr	17 v 1		13	0.0027 (0.0016, 0.0059, 0.0059)	0.28	19	0.062
chil v lyco	17 v 1			0.0027 (0.0016, 0.0059, 0.0059)	0.28		0.026
peru v ochr	17 v 1		15	0.0049 (0.0026, 0.0118, 0.0117)	0.22	43	0.185
peru v lyco	17 v 1			0.0049 (0.0026, 0.0118, 0.0117)	0.22		0.146
LYK3		1992					
chil v ochr	17 v 1		16	0.0046 (0.0011, 0.0160, 0.0159)	0.07	37	0.036
chil v lyco	17 v 1			0.0046 (0.0011, 0.0152, 0.0151)	0.07		0.718
peru v ochr	16 v 1		15	0.0056 (0.0008, 0.0211, 0.0209)	0.04	51	0.005
peru v lyco	16 v 1			0.0056 (0.0008, 0.0215, 0.0214)	0.04		0.822
LYK4		1938					
chil v ochr	15 v 1		15	0.0080 (0.0061, 0.0138, 0.0137)	0.44	51	0.510
chil v lyco	15 v 1			0.0080 (0.0061, 0.0138, 0.0137)	0.44		0.760
peru v ochr	16 v 1		16	0.0089 (0.0054, 0.0202, 0.0201)	0.26	80	0.275
peru v lyco	16 v 1			0.0089 (0.0054, 0.0202, 0.0201)	0.26		0.336
LYK6		1599					
chil v ochr	15 v 1		13	0.0151 (0.0119, 0.0246, 0.0246)	0.48	108	0.108
chil v lyco	15 v 1			0.0151 (0.0119, 0.0245, 0.0245)	0.48		0.069
peru v ochr	8 v 1		8	0.0161 (0.0135, 0.0244, 0.0244)	0.55	87	0.118
peru v lyco	8 v 1			0.0161 (0.0135, 0.0244, 0.0244)	0.55		0.064
LYK8		1149					
chil v ochr	13 v 1		12	0.0048 (0.0044, 0.0064, 0.0064)	0.69	34	0.025
peru v ochr	17 v 1		16	0.0052 (0.0042, 0.0087, 0.0087)	0.47	55	0.026
LYK9		1890					
chil v ochr	17 v 1		17	0.0073 (0.0042, 0.0174, 0.0176)	0.24	74	0.818
chil v lyco	17 v 1			0.0073 (0.0042, 0.0174, 0.0176)	0.24	73	0.604
peru v ochr	17 v 1		17	0.0068 (0.0045, 0.0135, 0.0146)	0.33	79	0.813
peru v lyco	17 v 1			0.0068 (0.0045, 0.0174, 0.0146)	0.33	77	0.649

Population samples were available for *Solanum chilense* (chil) and *Solanum peruvianum* (peru). Single allelic sequences from *Solanum ochranthum* (ochr) and *Solanum lycopersicoides* (lyco) were used for outgroup comparisons in the tests of neutrality. Haplotypes, the number of unique alleles in each population sample, apply to the first species listed in column 1, as do values for S, the number of segregating sites. The ratio of non-synonymous to synonymous average pairwise differences (*π*_*a*_/*π*_*s*_) was calculated for *S. chilense* and *S. peruvianum*. The McDonald-Kreitman test was applied to each pair of species listed in column 1. Uncorrected *p*-values for the McDonald-Kreitman analyses are reported, but following correction for multiple testing, none of the corrected p-values were below 5%

We identified six LysM-RLKs for which our criterium for a minimum sample size for complete gene sequences from 8 different individuals for both *S. chilense* and *S. peruvianum* was met. This corresponded to the following set of genes: *LYK1, LYK3, LYK4, LYK6, LYK8* and *LYK9*. The polymorphism at non-synonymous sites at the LysM-RLK genes ranged from 0.11% (*LYK3*) to 1.19% (*LYK6*) in *S. chilense* and 0.08% (*LYK3*) to 1.4% (*LYK6*) in *S. peruvianum* (Table 1). The polymorphism at synonymous sites at the LysM-RLK genes ranged from 0.64% (*LYK8*) to 2.5% (*LYK6*) in *S. chilense* and 0.87% (*LYK8*) to 2.4% (*LYK6*) in *S. peruvianum*. These values are consistent with the species-wide mean polymorphism at non-synonymous (0.18%) and synonymous (1.27%) sites in *S. chilense* and non-synonymous (0.22%) and synonymous (1.69%) sites in *S. peruvianum* as reported in [19].

To determine whether selection had differential effects on the pattern of sequence polymorphism in the intracellular or extracellular domains, we calculated the summary statistics for each of these domains separately (Table 2). The ratio of non-synonymous (*π*_*a*_) and synonymous (*π*_*s*_) pairwise differences is often used to gauge the impact of natural selection on the distribution of sequence variation [50]. For most genes, the ratio of *π*_*a*_/*π*_*s*_ was higher in the extracellular domain than in the intracellular/kinase domain. The distribution of variation at *LYK3* gene stands out because the ratios of *π*_*a*_/*π*_*s*_ are extremely low (≤ 0.06) and are nearly equivalent in the extracellular and intracellular domains (Table 2). This indicates that a similar degree of purifying selection may be acting on the intracellular and extracellular domains of *LYK3* in wild tomatoes.

**Table 2:** Summary of allelic diversity applied separately to extracellular and intracellular domains of LysM-RLKs from wild tomatoes

	Gene region	No. Seqs	No. Sites	Haplotypes	π all sites (non,syn,silent)	*π*_*a*_/*π*_*s*_	S
LYK1							
chil v ochr	Extracellular	17 v 1	699	8	0.0026 (0.0025, 0.0032, 0.0032)	0.79	8
	Intracellular		1101	9	0.0028 (0.0011, 0.0079, 0.0078)	0.14	10
chil v lyco	Extracellular	17 v 1	699	8	0.0026 (0.0025, 0.0032, 0.0032)	0.80	8
	Intracellular		1101	9	0.0028 (0.0011, 0.0079, 0.0078)	0.14	10
peru v ochr	Extracellular	17 v 1	699	12	0.0063 (0.0042, 0.0129, 0.0129)	0.33	21
	Intracellular		1101	15	0.0043 (0.0018, 0.0117, 0.0116)	0.16	21
peru v lyco	Extracellular	17 v 1	699	12	0.0063 (0.0043, 0.0130, 0.0130)	0.33	21
	Intracellular		1101	15	0.0043 (0.0018, 0.0117, 0.0116)	0.16	21
LYK3							
chil v ochr	Extracellular	17 v 1	705	12	0.0046 (0.0005, 0.0176, 0.0176)	0.03	13
	Intracellular		1203	16	0.0044 (0.0009, 0.0158, 0.0156)	0.06	21
chil v lyco	Extracellular	17 v 1	705	12	0.0046 (0.0005, 0.0155, 0.0155)	0.03	13
	Intracellular		1203	16	0.0044 (0.0009, 0.0158, 0.0156)	0.06	21
peru v ochr	Extracellular	16 v 1	705	13	0.0086 (0.0008, 0.0328, 0.0328)	0.03	24
	Intracellular		1203	14	0.0042 (0.0008, 0.0155, 0.0153)	0.05	25
peru v lyco	Extracellular	16 v 1	705	13	0.0088 (0.0009, 0.0349, 0.0349)	0.02	24
	Intracellular		1203	14	0.0042 (0.0008, 0.0155, 0.0153)	0.05	25
LYK4							
chil v ochr	Extracellular	15 v 1	801	15	0.0089 (0.0074, 0.0136, 0.0136)	0.54	18
	Intracellular		1053	14	0.0074 (0.0053, 0.0139, 0.0137)	0.38	31
chil v lyco	Extracellular	15 v 1	801	15	0.0089 (0.0074, 0.0136, 0.0136)	0.54	18
	Intracellular		1053	14	0.0074 (0.0053, 0.0139, 0.0137)	0.38	31
peru v ochr	Extracellular	16 v 1	801	16	0.0106 (0.0087, 0.0161, 0.0089)	0.54	41
	Intracellular		1053	11	0.0075 (0.0029, 0.0236, 0.0233)	0.12	33
peru v lyco	Extracellular	16 v 1	801	16	0.0106 (0.0087, 0.0161, 0.0161)	0.54	41
	Intracellular		1053	11	0.0075 (0.0029, 0.0236, 0.0233)	0.12	33
LYK6							
chil v ochr	Extracellular	15 v 1	780	12	0.0146 (0.0132, 0.0198, 0.0198)	0.66	29
	Intracellular		741	13	0.0150 (0.0105, 0.0265, 0.0265)	0.39	31
chil v lyco	Extracellular	15 v 1	780	12	0.0146 (0.0132, 0.0198, 0.0198)	0.66	29
	Intracellular		741	13	0.0150 (0.0105, 0.0265, 0.0265)	0.39	31
peru v ochr	Extracellular	8 v 1	780	8	0.0152 (0.0149, 0.0166, 0.0166)	0.90	33
	Intracellular		741	8	0.0188 (0.0127, 0.0397, 0.0397)	0.31	30
peru v lyco	Extracellular	8 v 1	780	8	0.0152 (0.0149, 0.0165, 0.0165)	0.90	33
	Intracellular		741	8	0.0188 (0.0127, 0.0397, 0.0397)	0.31	30
LYK8							
chil v ochr	Extracellular	13 v 1	771	9	0.0042 (0.0053, 0.0006, 0.0006)	9.1	9
	Intracellular*		300	3	0.0022 (0.0000, 0.0116, 0.0116)	0.00	3
peru v ochr	Extracellular	17 v 1	771	13	0.0038 (0.0032, 0.0058, 0.0058)	0.55	16
	Intracellular*		300	9	0.0047 (0.0035, 0.0101, 0.0101)	0.34	11
LYK9							
chil v ochr	Extracellular	17 v 1	774	14	0.0086 (0.0068, 0.0145, 0.0145)	0.47	22
	Intracellular		1038	14	0.0060 (0.0020, 0.0199, 0.0202)	0.10	22
chil v lyco	Extracellular	17 v 1	774	14	0.0086 (0.0068, 0.0145, 0.0145)	0.47	22
	Intracellular		1038	14	0.0060 (0.0020, 0.0199, 0.0202)	0.10	22
peru v ochr	Extracellular	17 v 1	774	14	0.0053 (0.0040, 0.0092, 0.0092)	0.44	25
	Intracellular		1038	17	0.0082 (0.0051, 0.0168, 0.0188)	0.30	32
peru v lyco	Extracellular	17 v 1	774	14	0.0053 (0.0040, 0.0092, 0.0092)	0.44	25
	Intracellular		1038	17	0.0082 (0.0052, 0.0169, 0.0189)	0.30	32

Population samples were available for *Solanum chilense* (chil) and *Solanum peruvianum* (peru). Single allelic sequences from *Solanum ochranthum* (ochr) and *Solanum lycopersicoides* (lyco) were used for outgroup comparisons. Haplotypes, the number of unique alleles in each population sample, apply to the first species listed in column 1, as do values for S, the number of segregating sites. The ratio of non-synonymous to synonymous average pairwise differences (*π*_*a*_/*π*_*s*_) was calculated for *S. chilense* and *S. peruvianum*. *The kinase domain of LYK8 is truncated

We then applied two standard tests of neutrality to determine if the pattern of genetic variation was consistent with a recent history of directional or balancing selection. According to Tajima’s D, no evidence for selection at these six genes could be detected. According to the McDonald-Kreitman test, three genes may have experienced recent selection: *Sl*LYK1, *Sl*LYK3, and *Sl*LYK8 (Table 1). In the analysis of alleles of *Sl*LYK3 from *S. peruvianum* (with *S. ochranthum* as the outgroup), an excess of non-synonymous fixed differences between species was observed. This is consistent with a recent bout of directional selection at this locus. However, after correcting for multiple testing, the corrected *p*-value exceeded a significance threshold of 0.05. After 22 tests, the Šidák correction [51] requires a p-value of 0.0023 or less for a 5% significance threshold. The lowest uncorrected p-value for an individual test – *Sl*LYK3 with *p* = 0.00507 – is equivalent to a p-value of 10–11% after correction. Therefore, following correction for multiple testing, we fail to reject the null hypothesis of equal ratios of replacement to silent changes for fixed differences compared to segregating sites. In summary, the intraspecific and interspecific ratios of non-synonymous and synonymous variation are consistently less than 1 for all the genes analyzed. This indicates the action of purifying selection operating at these loci. Furthermore, the tests of neutrality did not indicate strong balancing or directional selection operating at these loci, except possibly with the case of *Sl*LYK3, a member of the red clade.

### Purifying selection extends to some LysM-RLKs in *A. thaliana*

To see if similar evolutionary patterns were present at LysM-RLKs in other plants species, we extended our population genetic analysis to the model plant species, *A. thaliana*. We analyzed the sequence variation from 68 accessions for the following genes: *At*LYK1, *At*LYK2, *At*LYK3, *At*LYK4 and *At*LYK5 (Table 3). These genomic sequences were retrieved from Cao et al. [52]. In particular, we focused on the distribution of variation at *At*LYK3, since this gene is a member of the red clade (Fig. 1) and is phylogenetically closely related to *Sl*LYK3, which showed evidence for strong purifying selection in wild tomatoes (Tables 1 and 2). The patterns of variation at *At*LYK3 are also consistent with the action of purifying selection (Table 3: π_a_/π_s_ = 0.178). However, the ratio of π_a_/π_s_ is not as low as it is for *LYK3* in wild tomatoes (Table 1; π_a_/π_s_ ≤ 0.06).

**Table 3:** Summary of allelic diversity at LysM-RLK from *A. thaliana*

	π (silent)	π (non-coding)	π (syn)	π (non-syn)	*π*_*a*_/*π*_*s*_	π (all sites)
AtLYK1	0.01519	0.00745	0.04017	0.00285	0.069	0.00996
-extracellular	0.01687	0.00490	0.04678	0.00273	0.057	0.01017
-intracellular	0.01469	0.00847	0.03933	0.00177	0.044	0.00970
AtLYK2	0.00134	0.00290	0.00098	0.00081	0.820	0.00094
-extracellular	0.00242	0.00296	0.00211	0.00026	0.123	0.00097
-intracellular	0.00080	0.00000	0.00081	0.00082	1.015	0.00082
AtLYK3	0.01090	0.00932	0.01570	0.00281	0.178	0.00730
-extracellular	0.01258	0.00875	0.02217	0.00587	0.262	0.00939
-intracellular	0.01032	0.00956	0.01307	0.00121	0.092	0.00650
AtLYK4	0.00258	0.00273	0.00236	0.00188	0.794	0.00219
-extracellular	0.00236	0.00326	0.00057	0.00265	4.629	0.00251
-intracellular	0.00299	0.00195	0.00433	0.00075	0.173	0.00164
AtLYK5	0.00258	0.00325	0.00150	0.00061	0.403	0.00151
-extracellular	0.00153	0.00162	0.00136	0.00000	0.000	0.00077
-intracellular	0.00366	0.00507	0.00163	0.00110	0.672	0.00220

### Wild tomatoes encode orthologs of *Sl*LYK8 with intact kinase domains

In the annotated genome of the cultivated tomato, *S. lycopersicum*, the *SlLYK8* gene is predicted to encode an extracellular LysM region and a truncated intracellular kinase domain. The *SlLYK8* gene is lacking more than half of the kinase region present in the closely-related *SlLYK9* gene*.* The missing region includes the catalytic loop and activation segment of the kinase. However, we observed strong protein sequence conservation in the intracellular regions of the orthologs of *Sl*LYK8 in *S. peruvianum* and *S. chilense* (Table 2: π_a_/π_s_ = 0.34 and π_a_/π_s_ = 0.00 respectively). This was counter to our intuition that natural selection would be relaxed in the intracellular region, if these genes no longer encoded functional kinases. Alternatively, we reasoned that the *SlLYK8* gene may be intact in the wild species and only recently truncated (or mis-annotated) in the cultivated species, *S. lycopersicum*. To test this hypothesis, we evaluated the de novo assembled transcriptomes of these wild species (assembled without read-mapping to the annotated genome of *S. lycopersicum*). This was necessary because coding regions *not* annotated in the original *S. lycopersicum* reference genome would fail to be mapped from the wild species, even if they were present in these wild species.

**Fig. 5:**
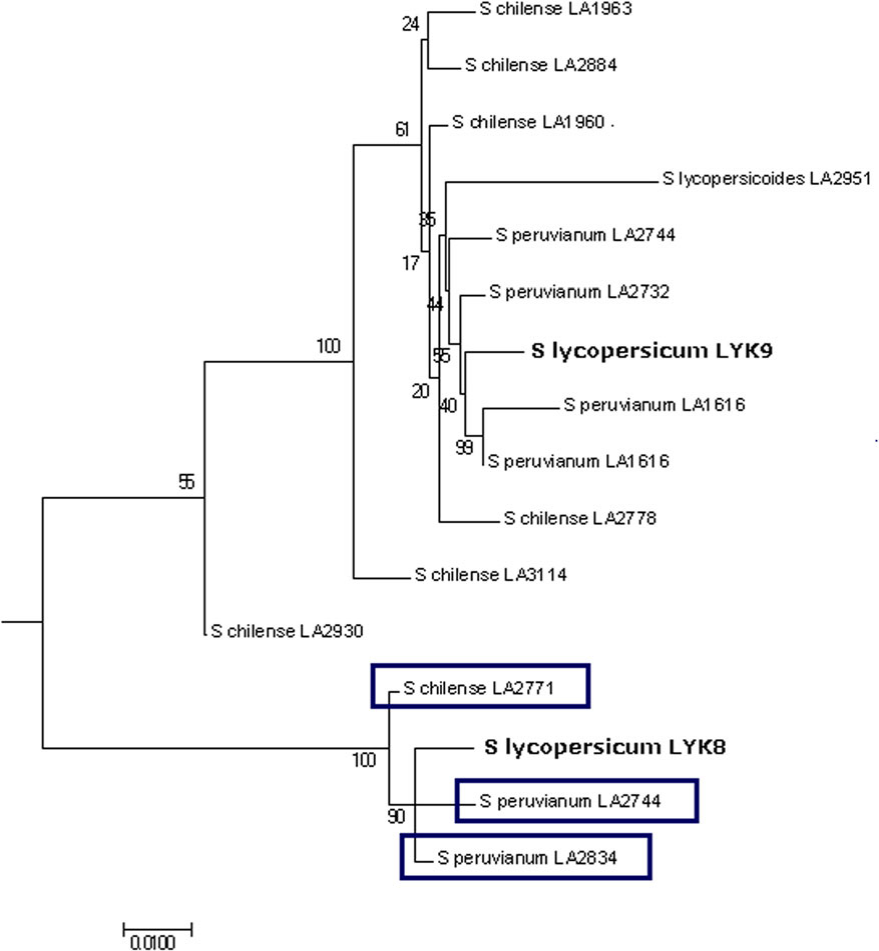
Phylogeny of wild tomato *Sl*LYK8 and *Sl*LYK9 orthologs with intact kinases. This phylogeny includes BLAST hits for *Solanum lycopersicum* LysM-RLKs *Sl*LYK8 and *Sl*LYK9 which extend past the point of *Sl*LYK8 truncation. The phylogeny and 500 bootstrap replicates were inferred using RAxML under the GTRGAMMA model and seed values of 100 and rooted using the method of Minimal Ancestor Deviation [27]. The scale bar indicates nucleotide substitutions per site. Three sequences from *Solanum peruvianum* and *Solanum chilense* with intact kinase domains share more recent ancestry with *Sl*LYK8 than with *Sl*LYK9 and show the greatest percent identity with *Sl*LYK8

We screened the de novo assemblies to check whether *SlLYK8* orthologous sequences were linked to kinase-encoding sequences [19]. Due to its high sequence similarity to *SlLYK8*, we also included *SlLYK9* in our analyses. The genomic sequences of *SlLYK8* and *SlLYK9* were used as query sequences in a BlastN search against the de novo assembled transcriptomes. After filtering by sequence length and percent identity to *SlLYK8* and *SlLYK9*, no single sequence was assigned to both queries. Amino acid translations of the sequences which extended beyond the original position of *Sl*LYK8 truncation in *S. lycopersicum* were aligned with MUSCLE, and this alignment was used to infer a maximum likelihood tree rooted using the method of MAD [27] (Fig. 5). Three sequences had higher sequence similarity to *Sl*LYK8 than to *Sl*LYK9 and extended beyond the position of *Sl*LYK8 truncation. Further inspection of the sequences revealed that each has an intact kinase, including all essential kinase residues. One of the full-length sequences was found in *S. chilense* and two were from *S. peruvianum*, which suggests that the truncation of the kinase in *Sl*LYK8 happened after the divergence between *S. lycopersicum* and the wild tomato species included here.

## Discussion

Here we investigated the evolutionary history of LysM-RLKs, with a special focus on wild tomatoes. These species show evidence of strong purifying selection at these genes, as opposed to directional or balancing selection observed at other well-known pathogenic recognition proteins in wild tomatoes [53–55]. In particular, the orthologs of *Sl*LYK3 have been subject to especially strong purifying selection, specifically in their extracellular domains. This gene belongs to the red clade of LysM-RLKs, which has distinct first and second LysM domains compared to other members of group I (Figs. 1 and 4).

There are currently no data on the function and/or specificity of *Sl*LYK3 or its orthologs from wild tomatoes, but its homolog, *At*LYK3, from *A. thaliana* negatively regulates fungal and bacterial defense [38] and is essential for suppressing the flg22-triggered immune responses in the presence of Nod factors [39]. The presence of strong protein conservation of *Sl*LYK3 orthologs within wild tomatoes, coupled with the distinctness of its LysM domains, makes it an interesting candidate for further functional characterization. Like *At*LYK3, orthologs in tomato may fulfill multiple roles. It may do this by detecting multiple microbial ligands with partner receptors or by coordinating signals to control immune response after microbe detection.

Our analyses also uncovered additional novel aspects of LysM-RLK evolution in tomatoes. For example, we detected complete kinase domains in orthologs of *Sl*LYK8 from *S. chilense* and *S. peruvianum*. While it is still unclear whether all wild tomatoes have orthologs of *Sl*LYK8 with intact kinase domains, its presence in both *S. peruvianum* and *S. chilense* shows that some wild tomatoes do. Sequences from *SlLYK8* alleles with intact kinases from other individuals may have been below our cutoffs for percent identity or sequence length or not sufficiently expressed at the time samples were taken. If intact kinase domains are found in additional wild tomato species, it would suggest that the truncation of the kinase may be unique to *S. lycopersicum* and could have been a fairly recent evolutionary change. Broader taxonomic sampling and analysis of genomic sequences will help to resolve the timing and direction of these evolutionary changes.

It was previously postulated that Group III originated after the split between Group I and Group II, because Group III is only found in dicots [11]. Our analysis indicates that the extracellular (LysM triplet) domain regions of Group II and Group III genes are more closely related to each other than either is to genes from Group I, and that the kinase domains of Group III genes differ from those of Groups II and III (as previously postulated [10, 11]). Despite expanding the known number of species containing LysM-III genes, we still did not find any outside of the Rosids, a clade of the Eudicots. Zhang et al. noted that LysM-II genes in both *M. truncatula* and *O. sativa* lacked activation loops and conservation at residues necessary for activity [6], and we note that the same is true for the Glycine-rich loop; this sequence is missing in every LysM-II gene in our analysis, and multiple representatives of this clade are present in each species. Taken together, this implies that the initial divergence in this gene family was between Group I and Group II LysM-RLKs, and that Group III LysM-RLKs originated subsequently (but prior to the divergence of *E. grandis* from the other Rosids) via a fusion of a Group II LysM triplet with a kinase domain from another protein family.

## Conclusions

The LysM-RLKs belong to a diverse family of proteins with many functions in plant symbiosis and defense and little correspondence between function and phylogenetic relationships. Here we provided an overview of the functions and phylogenetic relationships and found that the Group III LysM-RLKs share a closer relationship with those in Group II than those in Group I. Newly identified Group III LysM-RLKs were found in a variety of Rosid species. The kinase domain of *Sl*LYK8 homologs is intact in at least some wild tomato species. We suggest that *Sl*LYK3 is a prime candidate for future functional analysis, owing to its close relationship to the multi-functional *At*LYK3, its distinct LysM domains, and signs that the extracellular domains experience strong protein conservation in wild tomatoes.

## Methods

### Sources for protein sequences

Amino acid sequences from *A. thaliana*, *S. lycopersicum*, *L. japonicus*, *O. sativa*, and *M. truncatula* were obtained from the sources listed in Additional file 1: Table S1 [9–18]. To determine the positions of the three LysM domains, we identified the Cysteine residues (including CXC motifs) which occur prior to the start of the first LysM domain and between the proceeding LysM domains. The conserved Proline at the end of the third LysM domain marked the end of the LysM triplet.

### Transcriptome data and coverage selection criteria

DNA sequences of LysM-RLKs from *Solanum chilense*, *Solanum peruvianum*, *Solanum ochranthum*, and *Solanum lycopersicoides* were obtained from Beddows et al. [19], with the exception of the *Sl*LYK8 sequences, which were compiled from the same source data but with minimum read depth of 5 (Additional file 2). The study by Beddows et al. [19] provides an extensive transcriptome dataset for individuals of 18 different populations of *S. chilense* and *S. peruvianum*, respectively. In both studies, the two species, *S. ochranthum* and *S. lycopersicoides*, were used as outgroups. These two species are closely related to wild tomatoes, but lie outside the wild tomato clade. Because the cultivated tomato (*S. lycopersicum*) is nested within the clade of wild tomatoes [20], it could not be used as an outgroup. Sequences were included in the population genetic analysis, provided they met the following conditions for sequence completeness and sample size:

- Individual sequences had < 10% N-content (undetermined nucleotides) in the coding region
- A minimum sample size of 8 complete sequences (alleles) for both *S. chilense* and *S. peruvianum*. Alleles were sampled from different individuals independent of their gene sequence, i.e. alleles from different individuals may be identical in sequence.

Accession LA0752 was included in the *S. chilense* sequence set. Sequences from LA1274, an accession described as *Solanum corneliomulleri*, were included in the *S. peruvianum* data set (see Beddows et al. [19] for justification).

For the population genetic analysis of *Sl*LYK8, four genotypes (LA2750, LA2884, LA0752, and LA2930) were excluded because the alleles of *Sl*LYK8 could not be unambiguously inferred for these genotypes. De novo assembled transcriptomes were obtained from the same reads available from Beddows et al. [19]. They were assembled using Trinity [21] version 2014-07-17 with standard settings and mapped to *S. lycopersicum* (cultivar Heinz 1706) with GMAP [22] version 2017-05-08 with standard settings.

### Alignments

For data sets with fewer than 100 sequences or shorter than 200 amino acids, protein sequence alignments were done with the MUSCLE algorithm [24] implemented in MEGA7 [23]. For larger datasets, GUIDANCE [25] (with the MAFFT option) was used.

### Phylogenetic analyses

Phylogenies based on protein sequences were inferred in RAxML [26] using the protein substitution model that best fit the data (found using the PROTGAMMAAUTO function). For DNA sequences, the GTRGAMMA function was used. Branch support was assessed by performing 500 non-parametric bootstrap replicates. Seed values of 100 were chosen for reproducibility. Phylogenies were rooted with Minimal Ancestor Deviation (MAD) [27].

### Population genetic analysis

All population genetic tests were performed in DnaSP [28] on a randomly selected haplotype from each sampled individual. Significance for the McDonald-Kreitman test [29] was determined by the G-test when the assumptions of this test were met; otherwise, Fisher’s exact test was used. Tajima’s D [30] was calculated using the total number of mutations.

### The identification of *Sl*LYK8 from wild tomatoes

Two BlastN searches [31] were performed against the de novo transcriptomes described above: one with the *Sl*LYK8 sequence (1149 nucleotides in length) as the query and one with the corresponding positions of the *Sl*LYK9 sequence (1890 nucleotides in length) as the query. Percent identity was used to measure the similarity of the hits to *Sl*LYK8 and *Sl*LYK9. All hits at least 1000 nucleotides long and showing 97% or greater identity to either *Sl*LYK8 or *Sl*LYK9 (as reported in the BlastN results table) were included in further analyses. The sequences were translated in six frames and aligned together with the amino acid sequences from *Sl*LYK8 and *Sl*LYK9. Translations which spanned at least 40% of the *Sl*LYK8 gene and extended beyond the end of *Sl*LYK8 were included in the alignment.

### Identification of group III LysM-RLK homologs

The LjLYS20 amino acid sequence was used as a query in an online BlastP search (with max 250 hits and standard settings) [31] against NCBI’s non-redundant protein sequences database [32]. The full-length hits were aligned with the canonical LysM-RLKs and filtered according to the presence of the CXC motif between the first and second LysM domains. Exact duplicate sequences from the same species were removed before phylogenetic analysis.

## Additional files

Additional file 1: **Table S1.** Amino acid sequence sources. These are the sources of sequences used to infer the protein phylogenies [9–18]. (PDF 200 kb)

Additional file 2: *Sl*LYK8 ortholog sequences generated for this study. This is a Fasta-formatted text file containing the sequences generated from reads from several wild tomato species (*Solanum peruvianum*: peru, *Solanum chilense*: chil, *Solanum lycopersicoides*: lyco, and *Solanum ochranthum*: ochr) which were mapped to the region corresponding to *Sl*LYK8 in *Solanum lycopersicum*. Unlike the rest of the LysM-RLK orthologs obtained from [19], these sequences were assessed with a minimum read depth of five sequences. The rest of the mapping procedure was the same as that used for the other sequences. (TXT 35 kb)

Additional file 3: Phylogeny of new putative Group III LysM-RLKs and canonical LysM-RLKs. The maximum likelihood phylogeny and 500 bootstrap replicates were inferred using RAxML assuming the JTT model and seed values of 100. (PNG 987 kb)

Additional file 4: Phylogeny from Additional file 3 in Newick format. (NWK 7 kb)

Additional file 5: Phylogeny from Fig. 2a in Newick Format. (NWK 6 kb)

Additional file 6: Phylogeny from Fig. 2b in Newick Format. (NWK 6 kb)

Additional file 7: Phylogeny from Fig. 3 in Newick format. (NWK 6 kb)

Additional file 8: Phylogeny of reliably aligned individual LysM-RLK domains. Phylogeny of amino acid sequences of the LysM-RLK LysM domains which scored 0.80 or higher when evaluated with GUIDANCE. Each of the first domains scored below this cutoff, and all were omitted. All third domains were included. Second domains of genes highlighted in red and black were omitted. The maximum likelihood phylogeny and 500 bootstrap replicates were inferred using RAxML under the WAG model with empirical frequencies and seed values of 100. The Log-likelihood was − 9529. The second and third domains of the sequences included form distinct clades. Known functions of the proteins (Fig. 1) were mapped onto the individual domains in the tree. (PNG 1616 kb)

Additional file 9: Phylogeny from Additional file 8 in Newick format. (NWK 7 kb)

## Data Availability

The *Sl*LYK8 ortholog sequences generated for this study can be found in the Supplemental Material for this paper. All other sequences analyzed in this study can be found in public repositories as indicated in the citations.

## Abbreviations

*At*: *Arabidopsis thaliana*
CERK1: Chitin elicitor receptor-like kinase 1
*Eg*: *Eucalyptus grandis*
*Lj*: *Lotus japonicus*
LYK: LysM-RLK with classically conserved kinase domain
LYR: LYK-related, or LysM-RLK without classically conserved kinase domain
LYS: LysM-RLKs in *Lotus japonicus*
LysM-RLK: lysin motif RLK
*Mt*: *Medicago truncatula*
*Os*: *Oryza sativa*
RLK: receptor-like kinase
*Sl*: *Solanum lycopersicum*
