# Supplementary material for: The evolutionary history of LysM-RLKs (LYKs/LYRs) in wild tomatoes
Source: BMC Evol Biol. 2019 Jul 11;19:141. doi: 10.1186/s12862-019-1467-3 (PMC6625017; doi:10.1186/s12862-019-1467-3)
Supplement: Supplementary file 1 — Table S1. Amino acid sequence sources. These are the sources of sequences used to infer the protein phylogenies [9–18]. (PDF 200 kb) [file 12862_2019_1467_MOESM1_ESM.pdf]

Supplemental Table S1: Sources for protein sequences used in LysM-RLK phylogeny analysis

Name	Alt Name	Locus ID	GenBank	Source	Database
**AtLYK1**	AtCERK1	At3g21630		[12]	TAIR10 [13]
**AtLYK2**		At3g01840		[12]	TAIR10 [13]
**AtLYK3**		At1g51940		[12]	TAIR10 [13]
**AtLYK4**		At2g23770		[12]	TAIR10 [13]
**AtLYK5**		At2g33580		[12]	TAIR10 [13]
**LjNFR1**			CAE02589.1	[11]	[14]
**LjNFR5**			CAE02597.1	[11]	[14]
**LjLYS1**			AB503681	[11]	[14]
**LjLYS2**			AB503682	[11]	[14]
**LjLYS3**			AB503683	[11]	[14]
**LjLYS4**			AB503684	[11]	[14]
**LjLYS5**			AB503686	[11]	[14]
**LjLYS6**			AB503687	[11]	[14]
**LjLYS7**			AB503688	[11]	[14]
**LjLYS11**			AB503689	[11]	[14]
**LjLYS12**			AB503690	[11]	[14]
**LjLYS13**			AB503691	[11]	[14]
**LjLYS14**			AB503692	[11]	[14]
**LjLYS15**			AB503693	[11]	[14]
**LjLYS16**			AB503694	[11]	[14]
**LjLYS20**			AB503695	[11]	[14]
**LjLYS21**			AB503696	[11]	[14]
**MtNFP**	MtNFR5	Medtr5g019040.1		[10]	Mt4.0v2 [15]
**MtLYK1**		Medtr5g086540.1		[10]	Mt4.0v2 [15]
**MtLYK2**		Medtr5g086310.1		[10]	Mt4.0v2 [15]
**MtLYK3**		Medtr5g086130.1		[10]	Mt4.0v2 [15]
**MtLYK4**		Medtr5g086120.1		[10]	Mt4.0v2 [15]
**MtLYK5**		Medtr5g086090.1		[10]	Mt4.0v2 [15]
**MtLYK6**		Medtr5g086040.1		[10]	Mt4.0v2 [15]
**MtLYK7**		Medtr5g086030.1		[10]	Mt4.0v2 [15]
**MtLYK8**		Medtr2g024290.1		[10]	Mt4.0v2 [15]
**MtLYK9**		Medtr3g080050.1		[10]	Mt4.0v2 [15]
**MtLYK10**		Medtr5g033490.1		[10]	Mt4.0v2 [15]
**MtLYR1**		Medtr8g078300.1		[10]	Mt4.0v2 [15]
**MtLYR2**		Medtr1g021845.1		[10]	Mt4.0v2 [15]
**MtLYR3**		Medtr5g019050.1		[10]	Mt4.0v2 [15]
**MtLYR4**		Medtr5g085790.1		[10]	Mt4.0v2 [15]
**MtLYR5**		Medtr7g079350.1		[10]	Mt4.0v2 [15]
**MtLYR6**		Medtr7g079320.1		[10]	Mt4.0v2 [15]
**MtLYR7**		Medtr3g080170.1		[10]	Mt4.0v2 [15]
**OsLYK1**		LOC_Os01g36550		[12]	TIGR7 [16]

**OsLYK2**		LOC_Os06g41980		[12]	TIGR7 [16]
**OsLYK3**		LOC_Os06g41960		[12]	TIGR7 [16]
**OsLYK4**		LOC_Os02g09960		[12]	TIGR7 [16]
**OsLYK5**		LOC_Os03g13080		[12]	TIGR7 [16]
**OsLYK6**		LOC_Os11g35330		[12]	TIGR7 [16]
**SlLYK1**	Bti9	Solyc07g049180.2.1		[17]	ITAG2.4 [18]
**SlLYK2**		Solyc02g094010.1.1		[17]	ITAG2.4 [18]
**SlLYK3**		Solyc03g121050.2.1		[17]	ITAG2.4 [18]
**SlLYK4**		Solyc02g089900.1.1		[17]	ITAG2.4 [18]
**SlLYK6**		Solyc12g089020.1.1		[17]	ITAG2.4 [18]
**SlLYK7**		Solyc02g089920.1.1		[17]	ITAG2.4 [18]
**SlLYK8**		Solyc09g083200.2.1		[17]	ITAG2.4 [18]
**SlLYK9**		Solyc09g083210.2.1		[17]	ITAG2.4 [18]
**SlLYK10**		Solyc02g065520.1.1		[17]	ITAG2.4 [18]
**SlLYK11**		Solyc02g081040.2.1		[17]	ITAG2.4 [18]
**SlLYK12**		Solyc02g081050.2.1		[17]	ITAG2.4 [18]
**SlLYK13**		Solyc01g098410.2.1		[17]	ITAG2.4 [18]
**SlLYK14**		Solyc06g069610.1.1		[17]	ITAG2.4 [18]
**SlLYK15**		Solyc11g069630.1.1		[9]	ITAG2.4 [18]

All amino acid sequences analyzed in this study were obtained from publicly available databases as indicated in the database column. Citations (indicated in brackets) are available in the main text.
