# Supplementary figures and images for: The evolutionary history of LysM-RLKs (LYKs/LYRs) in wild tomatoes
Source: BMC Evol Biol. 2019 Jul 11;19:141. doi: 10.1186/s12862-019-1467-3 (PMC6625017; doi:10.1186/s12862-019-1467-3)

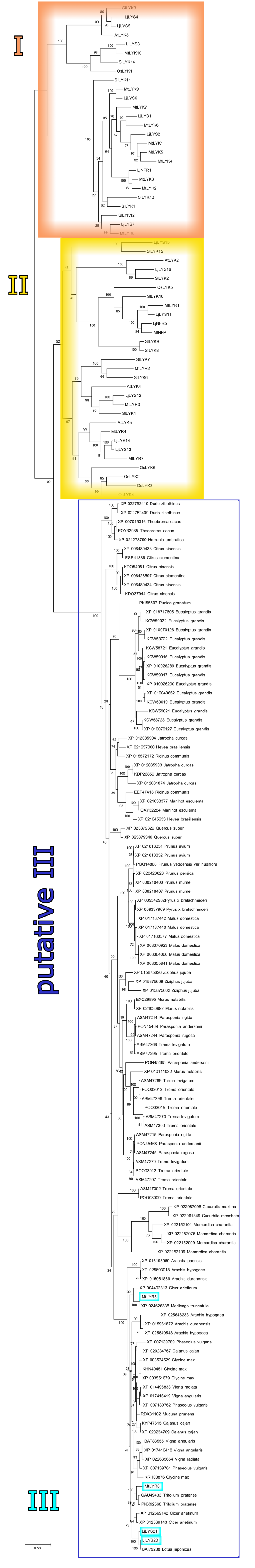

Supplement: Supplementary file 3 — Phylogeny of new putative Group III LysM-RLKs and canonical LysM-RLKs. The maximum likelihood phylogeny and 500 bootstrap replicates were inferred using RAxML assuming the JTT model and seed values of 100. (PNG 987 kb) [file 12862_2019_1467_MOESM3_ESM.png]

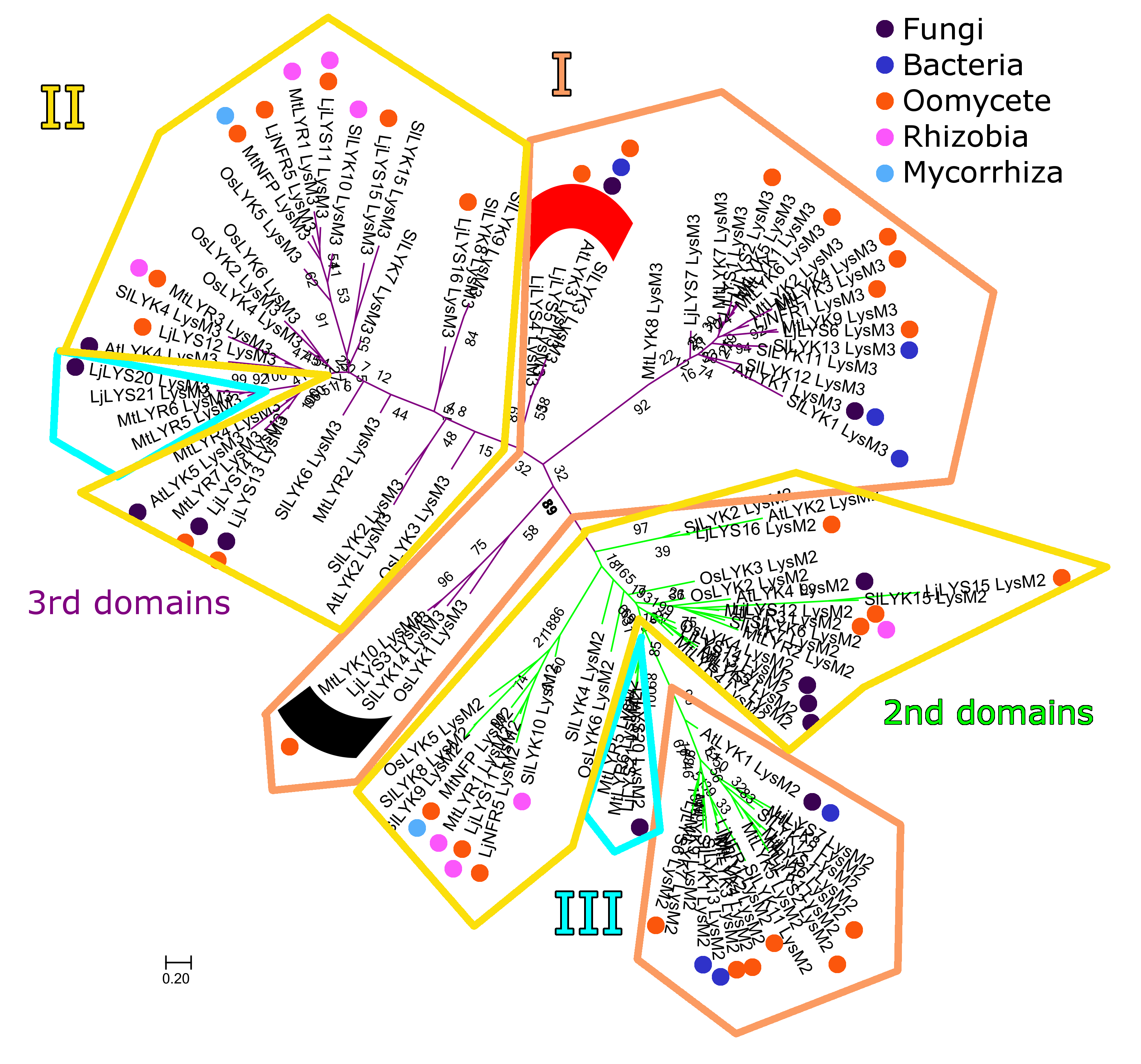

Supplement: Supplementary file 8 — Phylogeny of reliably aligned individual LysM-RLK domains. Phylogeny of amino acid sequences of the LysM-RLK LysM domains which scored 0.80 or higher when evaluated with GUIDANCE. Each of the first domains scored below this cutoff, and all were omitted. All third domains were included. Second domains of genes highlighted in red and black were omitted. The maximum likelihood phylogeny and 500 bootstrap replicates were inferred using RAxML under the WAG model with empirical frequencies and seed values of 100. The Log-likelihood was − 9529. The second and third domains of the sequences included form distinct clades. Known functions of the proteins (Fig. 1) were mapped onto the individual domains in the tree. (PNG 1616 kb) [file 12862_2019_1467_MOESM8_ESM.png]
